# Low-Temperature Spine-Specific PMMA Enhances Bone Regeneration via Localized Thermal Necrosis in an Osteoporotic Rat Model

**DOI:** 10.3390/ijms26104786

**Published:** 2025-05-16

**Authors:** Md Amit Hasan Tanvir, Md Abdul Khaleque, Ga-Hyun Kim, Sang-Eun Park, Hwan-Hee Lee, Young-Yul Kim

**Affiliations:** Department of Orthopedic Surgery, Daejeon St. Mary’s Hospital, The Catholic University of Korea, Seoul 34943, Republic of Korea; tanvir002@catholic.ac.kr (M.A.H.T.); abdulkhaleque.dream@gmail.com (M.A.K.); rlarkgus21@gmail.com (G.-H.K.); rockwood74@naver.com (S.-E.P.)

**Keywords:** spine-specific PMMA, biomaterials, osteoporosis

## Abstract

Poly (methyl methacrylate) (PMMA) bone cement is widely used in percutaneous vertebroplasty to stabilize osteoporotic vertebral compression fractures. However, its clinical application is limited by its high compressive modulus, risk of thermal necrosis, and poor bone integration, unlike conventional PMMA formulations used in vertebrae or joint arthroplasty, which can reach polymerization temperatures exceeding 100 °C. Spine-specific PMMA is formulated to cure at a reduced polymerization temperature, thereby minimizing the rise in core temperature during the setting process. Consistent with our hypothesis, this moderate thermal output induces localized thermal injury that triggers osteogenic responses and extracellular matrix production, thereby enhancing osteoblast activity in the surrounding bone. This study aimed to evaluate bone remodeling following spine-specific PMMA injection in an osteoporotic Sprague-Dawley (SD) rat model. Twenty-four osteoporotic female SD rats were randomly assigned to three groups: Control (untreated), OVX + spine-specific PMMA (OVX + PMMA), and OVX (OVX + Defect). Bone regeneration was assessed using dual-energy X-ray absorptiometry (DXA), micro-computed tomography (Micro-CT), quantitative PCR (qPCR), immunohistochemistry (IHC), and Western blotting. At 12 weeks post-injection, the OVX + PMMA group exhibited significantly greater bone regeneration than the OVX group. Micro-CT analysis demonstrated a marked increase in trabecular thickness in the PMMA-treated group. Notably, bone formation was more pronounced in regions surrounding the cement compared to adjacent untreated areas. This suggests that spine-specific PMMA promotes osteogenesis via localized thermal necrosis and subsequent osteoblast recruitment. These findings highlight the dual role of spine-specific PMMA in both structural stabilization and biologically driven bone regeneration. Further research is warranted to optimize its clinical applications while minimizing potential adverse effects.

## 1. Introduction

Osteoporosis is a widespread condition characterized by persistent bone pain and reduced muscle strength, impacting around 200 million individuals worldwide, particularly postmenopausal women [1]. As the global population grows older, the incidence of osteoporosis is steadily increasing [2]. Osteoporosis is a multifaceted systemic disorder marked by reduced bone density and mass, resulting in compromised bone microarchitecture, greater fragility, and an elevated risk of fractures and bone-related complications [3]. Vertebral compression fractures are a common consequence of osteoporosis, often referred to as osteoporotic vertebral compression fractures (OVCFs) [4]. Traditional approaches to managing pain from OVCFs, such as oral medications and immobilization, often fall short in delivering prompt relief and adequately reducing patient discomfort [5]. Consequently, minimally invasive techniques like percutaneous vertebroplasty (PVP) and balloon kyphoplasty (BKP) are commonly performed to stabilize and reinforce the vertebral body. These interventions typically involve the use of bone cements, with poly (methyl methacrylate) (PMMA) being the most widely applied material [6]. PMMA exhibits poor bioactivity and cannot be resorbed or substituted by natural bone over time. Consequently, the boundary between the bone and PMMA remains clearly defined, even after long-term implantation [7,8], which can lead to the loosening or displacement of the bone cement over time [9]. Therefore, some patients may need further surgical procedures. To overcome the limitations of PMMA-based bone cement, extensive research efforts have been dedicated to improving its performance. Over the past few decades, various approaches have been investigated, including the incorporation of natural bone powder [10], hydroxyapatite (HA) [11], linoleic acid, chitosan [12], and small intestinal submucosa [13]. In another study, the impact of incorporating α-tricalcium phosphate (α-TCP) and β-tricalcium phosphate (β-TCP) into PMMA bone cement was evaluated. While β-TCP had no effect on the mechanical strength, the addition of more than 3% α-TCP slightly reduced the compressive strength. Despite the regenerative benefits of TCP, higher concentrations may compromise the structural integrity of PMMA [14]. In the present study, the mechanical effects of adding HA particles of two grain sizes (5 µm and 10 µm) to commercially available PMMA bone cement were assessed at concentrations ranging from 0–10% dry mass. All samples were subjected to compressive testing to simulate the typical loading conditions experienced post-implantation. The results indicated that only the 2% HA addition, regardless of the particle size, significantly affected the mechanical properties of PMMA, whereas all other concentrations exhibited no measurable impact [15]. Additionally, this study evaluated the influence of glassy carbon (GC) particle incorporation into commercial PMMA bone cement at concentrations of 1–10% (*w*/*w*) and particle sizes of 0.4–12 μm and 20–50 μm. Compressive testing demonstrated that the addition of larger GC particles (20–50 μm) significantly reduced PMMA’s compressive strength, primarily due to polymerization disruption caused by the thermal characteristics of GC. Conversely, smaller GC particles (0.4–12 μm) had no significant effect on the mechanical properties across the tested concentration range [16]. However, many of these modifications present challenges, as the materials often fail to meet surgical requirements or exhibit insufficient compressive strength [17]. JH Tan et al. demonstrated that blood contamination during the preparation or application of PMMA bone cement can adversely affect its biomechanical strength by interfering with the polymerization process and reducing cement–bone interfacial integrity [17]. To minimize this risk, several intraoperative precautions should be implemented, including thorough saline irrigation to clear the surgical site, careful drying of the bone bed using sterile gauze or suction, changing gloves before handling the cement, and promptly inserting the implant after cement application. Blood contamination has been shown to increase porosity within the cement matrix, potentially weakening its mechanical properties and compromising implant fixation [18].

Historically, conventional PMMA used in vertebrae, knee, and hip arthroplasty generated excessive heat during polymerization, reaching temperatures over 100 °C, which led to spinal injuries and nerve damage and thermal necrosis, particularly in cases of cement leakage [19,20]. In response, spine-specific PMMA formulations have been developed to control exothermic reactions and reduce the peak polymerization temperature to below 100 °C [21].

To address these limitations, spine-specific PMMA formulations have been developed to achieve a lower peak polymerization temperature, thereby reducing the risk of thermal injury. Unlike traditional PMMA used in joint arthroplasties, spine-specific PMMA is optimized for vertebral applications, offering safer thermal profiles and potentially improved biocompatibility.

Recent studies have suggested that controlled thermal stress may not only minimize the cytotoxic effects but also stimulate bone remodeling through osteoblast activation. Mild heat stress has been shown to induce the release of heat shock proteins and modulate signaling pathways such as ERK and Wnt, contributing to osteogenic differentiation and matrix mineralization [22]. However, the therapeutic potential of spine-specific PMMA as a bioactive material, rather than a passive structural filler, remains underexplored

The exact mechanisms by which heat stress enhances osteogenesis remain unclear; however, studies using human MSCs have reported that Heat Shock Protein (HSP70) may play a role in promoting osteogenic activity [23]. HSP70 has been shown to enhance ALP activity and promote mineralization in human mesenchymal stem cells (hMSCs), while also significantly upregulating key osteogenic genes such as Runx2 and osterix. Both the current and previous studies [24] observed increased ALP expression and a higher number of TRAP-positive cells in the hyperthermia group, aligning with the findings reported by Chen et al. [23]. Another potential mechanism proposed by Li et al. is that heat exposure stimulates angiogenesis, which may contribute to enhanced bone regeneration [25].

In this study, we sought to investigate whether spine-specific PMMA induces a localized thermal effect that could paradoxically stimulate osteogenesis in osteoporotic bone. Using an ovariectomized (OVX) Sprague–Dawley rat model, we examined bone remodeling around the cement interface via histological, molecular, and imaging analyses. We hypothesized that spine-specific PMMA induces localized thermal necrosis, which subsequently promotes osteogenesis in osteoporotic bone.

## 2. Results

### 2.1. Spine-Specific PMMA Promotes Bone Formation in Ovariectomy-Induced Osteoporosis In Vivo

**Figure 1:**
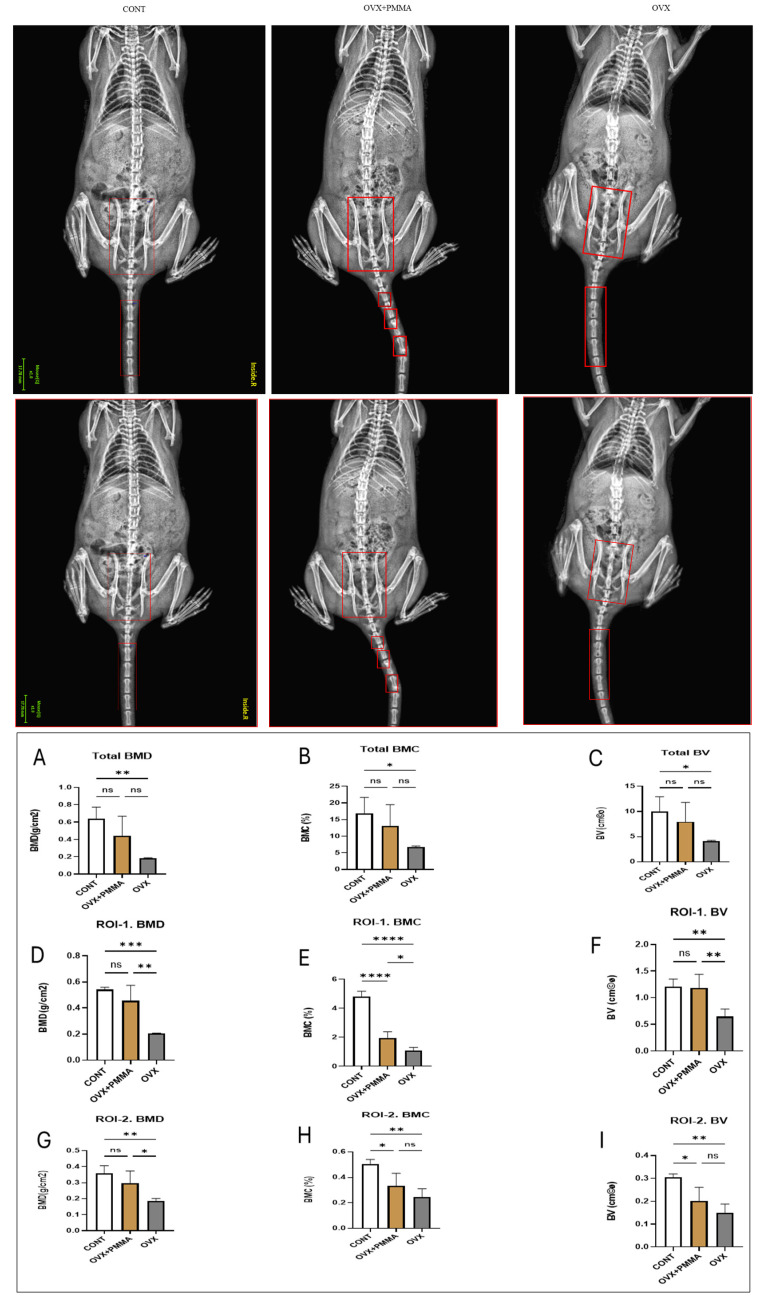
Panels (**A**–**C**) illustrate the bone mineral density (BMD) across the three groups, where the control group exhibits the highest values, the OVX group shows a significant reduction, and the OVX + Spine-Specific PMMA group demonstrates partial recovery following treatment. Panels (**D**–**F**) depict the bone mineral content (BMC) in ROI-1 (following a similar trend, with OVX-induced bone loss and subsequent improvement in the OVX + Spine-Specific PMMA group. Panels (**G**–**I**) represent bone volume (BV) in ROI-2, showing a marked decrease in the OVX group compared to the control, while spine-specific PMMA treatment mitigates bone loss, resulting in higher BV compared to the OVX group. (level of significance is * *p* < 0.05, ** *p* < 0.01, and *** *p* < 0.001, and **** *p* < 0.0001) (ns: no significance). (Red circles (ROI-2) indicate caudal vertebrae 3, 4, and 5. In this context, the control group remains untreated, the spine-specific PMMA group received cement injection, and the OVX group exhibits bone defects).

Bone mineral density (BMD), bone mineral content (BMC), and bone volume (BV) were measured in three different regions across the Control, OVX, and OVX + Spine-Specific PMMA groups (Figure 1). The control group exhibited normal bone growth across all parameters, serving as a baseline. In the OVX + Spine-Specific PMMA group, significant improvements were observed following 12 weeks of PMMA treatment after ovariectomy, indicating a partial restoration of bone properties. In contrast, the OVX group showed a substantial decline in BMD, BMC, and BV compared to the OVX +Spine-Specific PMMA group, reflecting osteoporosis-related bone loss. Our findings suggest that ovariectomy leads to a progressive reduction in bone quality, while spine-specific PMMA treatment facilitates a gradual improvement in bone parameters, highlighting its potential therapeutic role in osteoporosis management.

In the control group, baseline levels of ALP, RUNX2, and OCN were observed, indicating normal bone homeostasis. In the OVX group, a significant reduction in gene expression was noted for all three markers, consistent with osteoporosis-induced suppression of osteogenesis. In contrast, the OVX + Spine-Specific PMMA group demonstrated a marked upregulation of ALP, RUNX2, and OCN compared to the OVX group. Interestingly, expression levels in the OVX +Spine-Specific PMMA group were higher than those in the control group, suggesting that spine-specific PMMA biomaterials effectively stimulate osteogenic activity, even in osteoporotic conditions.

The results indicate that spine-specific PMMA biomaterials promote osteoblast activity and differentiation, as evidenced by the upregulation of ALP, RUNX2, and OCN. ALP serves as an early marker of osteoblast differentiation, reflecting the initial stages of bone matrix deposition, while RUNX2 is a critical transcription factor essential for osteoblast lineage commitment and differentiation. OCN, on the other hand, is a late-stage marker indicative of mature osteoblast function and bone formation.

The significantly higher gene expression levels in the OVX + Spine-Specific PMMA group compared to the OVX group highlight the therapeutic potential of PMMA biomaterials in mitigating the effects of osteoporosis. The observed upregulation suggests that spine-specific PMMA may enhance the bone microenvironment, thereby promoting osteogenesis and potentially reversing osteoporosis-induced impairments.

### 2.2. Spine-Specific PMMA Induces Bone Microarchitecture in OVX Rat

**Figure 2:**
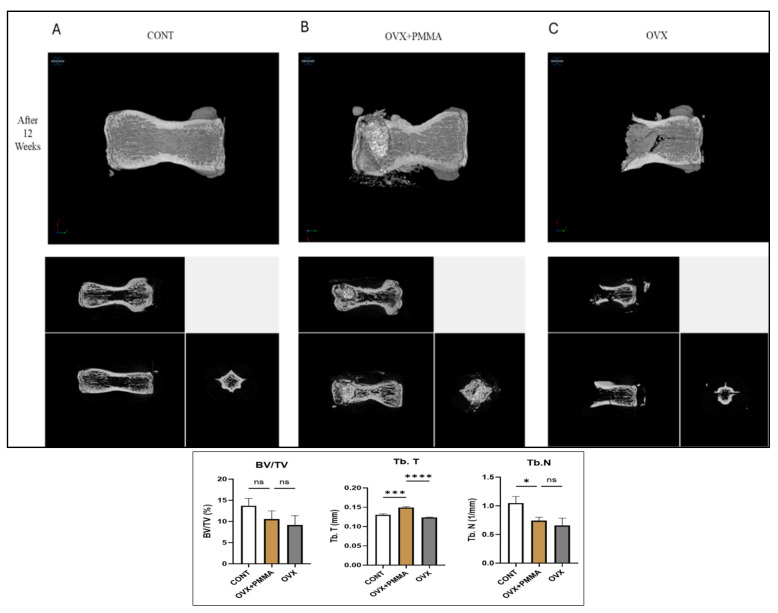
Micro-CT analysis of bone microarchitecture across experimental groups. (**A**) Representative 3D reconstruction of the control group, showing well-preserved trabecular architecture with high BV/TV, Tb.T, and Tb.N. (**B**) OVX + Spine-Specific PMMA group, demonstrating partial preservation of bone microarchitecture, with increased BV/TV, Tb.T, and Tb.N compared to the OVX group (**C**) OVX group, displaying significant bone loss, with reduced BV/TV, Tb.T, and Tb.N, indicating trabecular deterioration. (Level of significance is * *p* < 0.05, *** *p* < 0.001, and ****, *p* < 0.0001) (ns: no significance).

**Figure 3:**
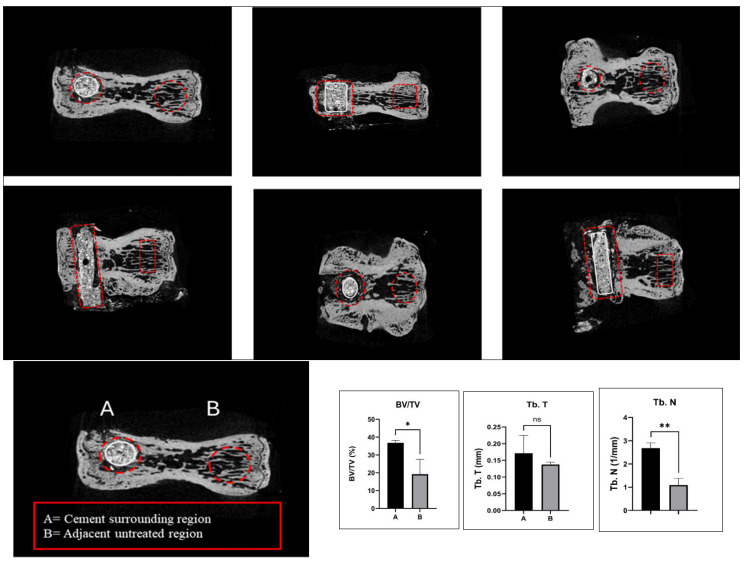
Micro-CT Analysis of cement-surrounding and adjacent untreated regions (**A**) representative micro-CT Images: Micro-CT images of bone samples showing structural differences between the cement-surrounding region (**A**) and the adjacent untreated region (**B**). The red dashed lines indicate the analyzed regions. (**B**) Quantitative analysis of bone microarchitecture: BV/TV is significantly higher in region A compared to region B (*p* < 0.05). For Tb. T, there is no significant difference between the two regions (ns). Tb. N is significantly greater in region A than in region B. (level of significance is * *p* < 0.05 and ** *p* < 0.01) (ns: no significance). (Region A: Cement surrounding region and Region B: Adjacent untreated region).

Micro-computed tomography illustrates distinct differences among groups. The control group exhibits a well-maintained trabecular structure, characterized by high bone volume fraction (BV/TV), trabecular thickness (Tb.Th), and trabecular number (Tb.N) (Figure 2A). The OVX + spine-specific PMMA group displays partial preservation of trabecular architecture, with improvements in BV/TV, Tb.Th, and Tb.N (Figure 2B). The OVX group shows pronounced bone loss, reflected by decreased BV/TV, Tb.Th, and Tb.N, indicating severe deterioration of the trabecular network (Figure 2C). Micro-CT analysis revealed a decline in bone volume fraction (BV/TV), trabecular thickness (Tb.T), and trabecular number (Tb.N) in the OVX group compared to the control and OVX + PMMA groups. However, BV/TV, Tb.T, and Tb.N were all elevated in the OVX + PMMA group relative to the OVX group, indicating partial preservation of the bone structure. Although the spine-specific PMMA treatment did not entirely prevent OVX-induced bone loss, further examination of the cement-surrounding and adjacent untreated regions showed a higher BV/TV, Tb.T, and Tb.N in the cement-surrounding region compared to the adjacent untreated region (Figure 3). These findings suggest that spine-specific PMMA injections contributed to an improvement in bone microarchitecture in both the OVX + Spine-Specific PMMA group and the cement-surrounding region, while the adjacent untreated region exhibited lower structural integrity.

### 2.3. Spine-Specific PMMA Mitigates OVX-Induced Bone Loss by Enhancing the Expression of Genes Associated with Bone Formation

**Figure 4:**
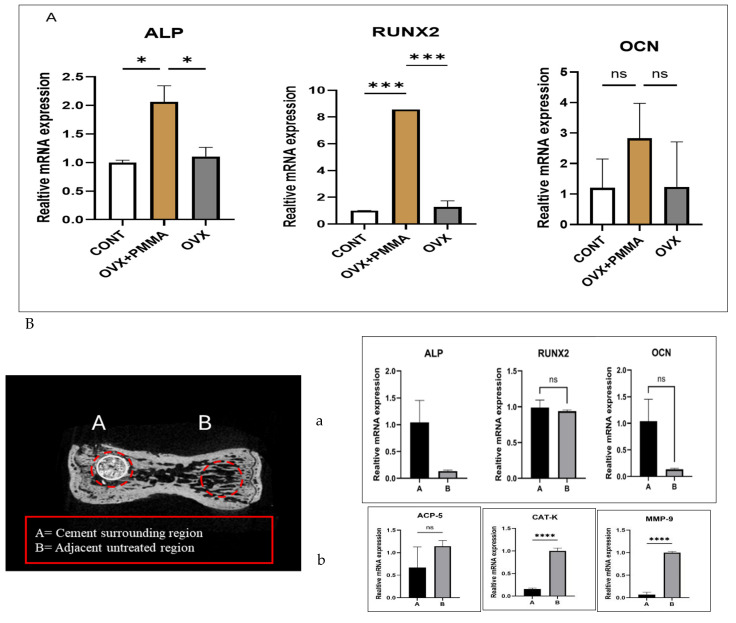
Gene expression analysis in the caudal vertebrae and cement-surrounding region. (**A**) Quantitative polymerase chain reaction (qPCR) analysis of osteogenic markers (ALP, RUNX2, and OCN) in the full caudal vertebrae, indicating overall gene expression levels. (**B**) qPCR analysis comparing gene expressions in the cement-surrounding region (**A**) and the adjacent untreated region (**B**). (**B**-**a**) The cement-surrounding region exhibits significantly higher ALP and OCN expression compared to the adjacent untreated region, while RUNX2 expression shows no significant difference. These findings suggest localized osteogenic activity in response to the cement material. (**B**-**b**) This figure represents the osteoclast activity in different experimental groups by analyzing the relative mRNA expression of key osteoclastic markers. ACP-5, CAT-K, and MMP-9 are significantly upregulated in group B compared to group A. These results suggest increased osteoclast activity in group B, implying higher bone resorption. (level of significance is * *p* < 0.05, *** *p* < 0.001, and ****, *p* < 0.0001) (ns: no significance).

The OVX + PMMA group showed the highest expression levels compared to the Control and OVX groups after 12 weeks of treatment. In the meantime, the OVX group showed higher osteoblast activity compared to the Control group. This was because of the ovariectomy. After ovariectomy, the body system tries to recover. The spine-specific PMMA injection into the caudal vertebrae initially caused thermal necrosis, which, in turn, stimulated osteoblast activation in the OVX + PMMA group. As predicted, this thermal necrosis triggered the bone remodeling process, leading to increased osteoblast activity. These findings suggest that spine-specific PMMA not only enhances mechanical stability but also induces a biological response that promotes bone formation, thereby helping to counteract OVX-induced bone loss (Figure 4A). Additionally, when assessing osteoblast activity in both the cement-surrounding and adjacent untreated regions, we observed significantly higher osteoblast activity in the thermally affected area compared to the adjacent untreated region, further supporting the role of spine-specific PMMA in enhancing bone regeneration (Figure 4B-a). The relative mRNA expression levels of key osteoclastic markers, including ACP-5, CAT-K, and MMP-9, were analyzed to assess the osteoclast activity in different experimental groups. As shown in the figure, the expression of ACP-5, a marker of osteoclast differentiation, was significantly higher in group B compared to group A (*p* < 0.05). Similarly, CAT-K, a crucial enzyme involved in bone matrix degradation, showed a significant increase in group B, indicating enhanced osteoclastic activity. Furthermore, MMP-9, a metalloproteinase associated with extracellular matrix remodeling and osteoclast function, was also significantly upregulated in group B compared to group A (*p* < 0.05). These findings suggest that group B exhibits an increased osteoclast-mediated bone resorption compared to group A (Figure 4B-b).

### 2.4. Spine-Specific PMMA Induces Osteoblast Activity in Rat Caudal Vertebrae

**Figure 5:**
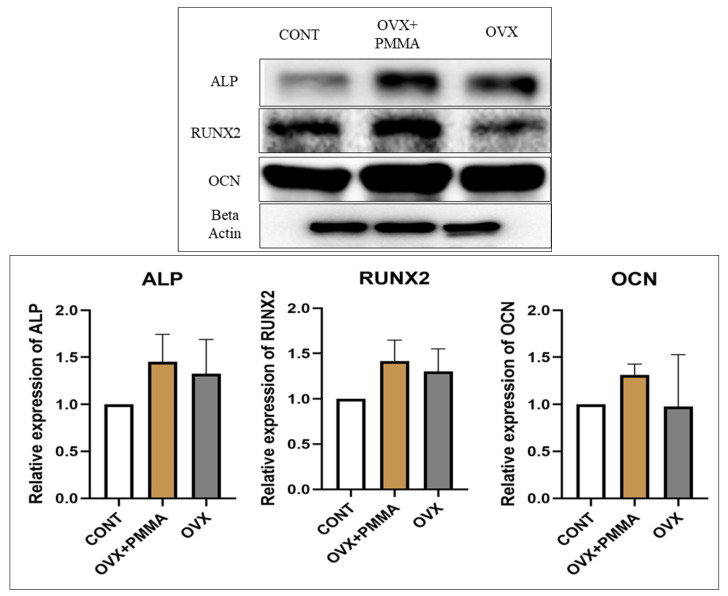
The expression of the ALP, RUNX2, and OCN protein among the Control, OVX+ Spine-Specific PMMA, and OVX groups. The Control group exhibits the baseline value, while the OVX group shows a reduction in osteoblast activity, indicating the effects of ovariectomy. Treatment with spine-specific PMMA in the OVX+ Spine-Specific PMMA group resulted in an increase in ALP, RUNX2, and OCN compared to the OVX group, suggesting a potential restorative or mitigating effect of spine-specific PMMA. Data are presented as mean ± standard deviation.

**Figure 6:**
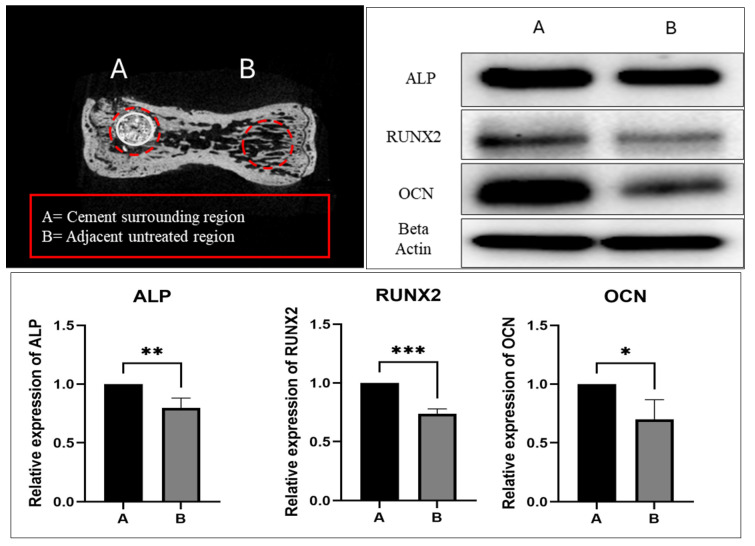
Relative expression levels of ALP, RUNX2, and OCN in groups A and B. No significant difference is observed in ALP expression between the two groups. However, the expression levels of RUNX2 and OCN are significantly lower in group B compared to group A (*p* < 0.01), as indicated by the asterisks. Data are presented as mean ± standard deviation. (level of significance is * *p* < 0.05, ** *p* < 0.01, and *** *p* < 0.001).

To evaluate osteoblast activity, we performed Western blot analysis for alkaline phosphatase (ALP), Runt-related transcription factor 2 (RUNX2), and osteocalcin (OCN). Our findings revealed a significant increase in osteoblast activity in the OVX+ Spine-Specific PMMA group compared to both the Control and OVX groups in 12 weeks. We hypothesized that the PMMA injection induces thermal necrosis, which subsequently stimulates osteoblast-related protein expression in the rat caudal vertebrae (Figure 5). To further investigate this, we analyzed both the surrounding cement region and the adjacent untreated region. The results confirmed that the region surrounding the cement exhibited elevated ALP, RUNX2, and OCN expression, supporting the hypothesis that thermal necrosis may trigger osteoblast activation as a compensatory bone remodeling response (Figure 6).

**Figure 7:**
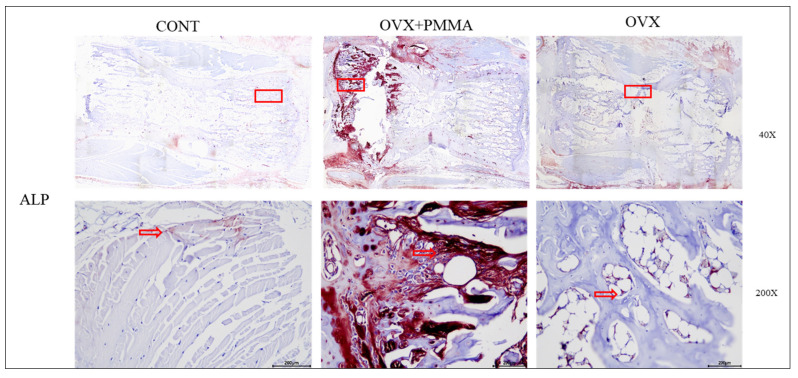
Histological expression levels in different experimental groups after 12 weeks of treatment. ALP expression was higher in the spine-specific PMMA group compared to both the Control and OVX groups, indicating enhanced osteogenic activity. (Red boxes indicate regions of interest that were further magnified in the 200× images below, and arrows point to positive ALP staining regions).

**Figure 8:**
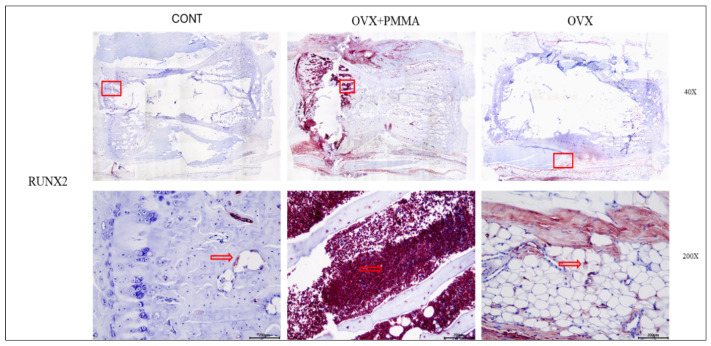
Histological expression levels of RUNX2 in different experimental groups after 12 weeks of treatment. RUNX2 expression was significantly elevated in the spine-specific PMMA group relative to the Control and OVX groups, suggesting increased osteoblast differentiation. (Red boxes indicate regions of interest that were further magnified in the 200× images below, and arrows point to positive RUNX2 staining regions).

**Figure 9:**
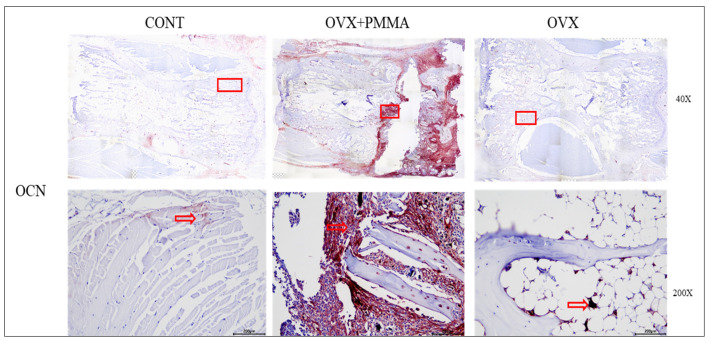
Histological expression levels of OCN in different experimental groups after 12 weeks of treatment. OCN level followed a similar trend, with the Spine-Specific PMMA group exhibiting greater expression than the Control and OVX group, further supporting enhanced bone formation. (Red boxes indicate regions of interest that were further magnified in the 200× images below, and arrows point to positive OCN staining regions).

**Figure 10:**
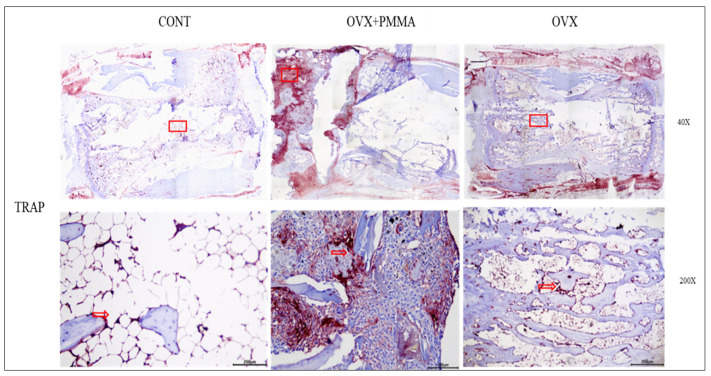
Representative images of TRAP (tartrate-resistant acid phosphatase) staining were obtained from the Control, OVX + PMMA, and OVX groups to evaluate osteoclast activity. In the Control group, minimal TRAP-positive staining was observed, indicating baseline osteoclast activity associated with normal bone turnover (top left and bottom left panels). In contrast, the OVX group exhibited extensive TRAP-positive staining along the trabecular surfaces, confirming elevated osteoclast numbers and activity, which is characteristic of estrogen-deficiency-induced bone resorption (top right and bottom right panels). Interestingly, the OVX + PMMA group demonstrated moderate TRAP activity. (Red boxes indicate regions of interest that were further magnified in the 200× images below, and arrows point to positive TRAP staining regions).

Additionally, to corroborate these findings at the tissue level, we conducted immunohistochemical (IHC) analysis, which revealed a similar trend—osteoblast activity was significantly higher in the OVX+ Spine-Specific PMMA group. Notably, osteoblast activity was predominantly elevated in the surrounding cement region, further supporting our hypothesis. This suggests that thermal necrosis induced by spine-specific PMMA may act as a microenvironmental stimulus, activating osteoblast-mediated bone remodeling as part of the bone’s adaptive response to localized damage (Figure 7, Figure 8 and Figure 9). To investigate the osteoclast activity in response to PMMA treatment, TRAP staining was performed on vertebral bone sections from the Control, OVX, and OVX + PMMA groups. In the Control group, only minimal TRAP-positive cells were observed, reflecting baseline osteoclast activity that is typical of normal bone homeostasis. As expected, the OVX group exhibited markedly increased TRAP staining, indicating heightened osteoclast presence and bone resorption activity due to estrogen deficiency. In contrast, the OVX + PMMA group showed moderate TRAP staining, primarily localized around the cement–bone interface. This suggests that PMMA administration does not provoke excessive osteoclastic activity and may instead contribute to a more balanced remodeling environment. These observations support the hypothesis that spine-specific PMMA implantation can modulate osteoclast behavior in osteoporotic bone (Figure 10).

## 3. Discussion

Vertebral compression fractures (VCFs) caused by osteoporosis are commonly seen in the elderly population [26]. Treatment of osteoporotic vertebral compression fractures (OVCFs) involves both traditional methods, such as oral medications and immobilization, as well as surgical options [9]. In recent years, vertebral augmentation and stabilization techniques, including PVP and BKP, have become increasingly common to improve treatment outcomes and reduce patient discomfort associated with OVCFs [27]. PMMA bone cement is widely used in vertebral prostheses, but it has two major limitations: a high elastic modulus and the absence of biological activity, both of which restrict its effectiveness and broader application [28].

Several studies have investigated the strategy of pre-cooling either the prosthesis or the femoral canal before implantation to reduce the peak temperature generated during PMMA bone cement polymerization. Research by Dipisa et al., Hsieh et al., and Rodop et al. has shown that this method effectively reduces thermal output, thereby minimizing the risk of heat-induced tissue damage. [29,30]. Their findings indicated that low-temperature PMMA cement generates less heat during polymerization, minimizing the risk of damage to surrounding bone tissue. Consequently, spine-specific PMMA cements are formulated to produce reduced exothermic reactions, lowering the chances of thermal necrosis. This milder thermal profile helps maintain osteoblast viability, thereby supporting bone regeneration and enhancing integration at the implant site.

Thermal stress during orthopedic procedures has been linked to osteocyte apoptosis, but its direct impact on bone remodeling is not fully understood. This study demonstrates that brief heat exposure (47 °C for 1 min) induces apoptosis in MLO-Y4 osteocyte-like cells and alters the expression of key osteogenic genes. A consistent downregulation of the Rankl/Opg ratio and upregulation of Cox2 indicate a shift toward osteoblastogenic signaling. Additionally, secreted factors from heat-treated osteocytes enhanced alkaline phosphatase activity and calcium deposition in mesenchymal stem cells. These findings suggest that thermally damaged osteocytes trigger a remodeling cascade, transitioning from early osteoclastogenic to later osteoblastogenic responses [31]. We hypothesize that exposure to mild thermal elevations generates moderate heat in the surrounding cement region, which subsequently promotes osteoblastic activity.

A recent study showed that applying mild heat stress (45 °C for 15 min, once weekly) to clinically approved materials (Resovist^®^ and REGENOS^®^) significantly enhanced bone formation in rat and rabbit defect models. Micro-CT and histological analyses revealed increased mineralized bone and the presence of osteoclasts in the heat-treated groups. In vitro, heat stress also increased alkaline phosphatase activity in osteoblastic cells but had no effect on chondrogenic differentiation. These results suggest that heat therapy could be a promising treatment for promoting osteogenesis in bone defect conditions [32]. Several studies involving human mesenchymal stem cells (MSCs), bone marrow stromal cells, and MG63 cells have demonstrated that heat stress in vitro promotes osteoblast differentiation and proliferation, boosts alkaline phosphatase (ALP) activity, and upregulates osteogenic markers [33]. Olkku et al. suggested that ultrasound-induced temperature increases could activate Wnt signaling, while other studies have indicated that elevated levels of heat shock proteins (HSPs) may also contribute to enhancing osteogenesis [34].

Osteoclasts are large, multinucleated cells derived from the monocyte/macrophage lineage, and their dynamic cytoskeletal structure allows for rapid morphological changes in response to bone-resorbing activity and environmental stimuli. In contrast, osteoblasts originate from mesenchymal stem cells and are typically small, mononuclear cells that form tight intercellular junctions. Their morphology remains relatively stable due to their adherence to the extracellular matrix during bone formation. Following treatment with spine-specific polymethyl methacrylate (PMMA), a noticeable shift in cellular activity is observed—osteoclast activity tends to decrease, likely due to the altered local environment and inhibition of bone resorption signaling pathways, whereas osteoblast activity increases, promoting new bone formation and enhancing the regenerative microenvironment.

In our study, we utilized the same spine-specific PMMA developed by Ahn D.K. et al. The polymerization temperature was reduced to 47.5 °C, which is significantly lower than the 74.2 °C observed in conventional PMMA, indicating a less intense exothermic reaction during the curing process. Furthermore, the setting time was shortened to 606 s, improving the material’s handling characteristics and minimizing the intraoperative waiting time [35].

This study demonstrates that spine-specific PMMA not only provides mechanical stabilization but also elicits biological effects conducive to bone regeneration in osteoporotic vertebrae. Unlike conventional PMMA, which generates excessive heat and lacks biological activity, the spine-specific formulation offers a more favorable thermal profile. This moderate heat may act as a localized stimulus, promoting osteogenesis.

Our findings align with prior reports indicating that mild heat stress can upregulate the osteogenic signaling pathways, such as those involving ERK, COX-2, and heat shock proteins. Moreover, in vivo studies have shown that periodic thermal exposure enhances bone formation without adversely affecting surrounding tissues [23]. Our qPCR, Western blot, and IHC data collectively support the notion that spine-specific PMMA induces a microenvironment favorable to osteoblast recruitment and activity. This study presents a compelling case for spine-specific PMMA as a dual-functional biomaterial.

Despite its valuable findings, this study has certain limitations. The use of rat caudal vertebrae does not accurately replicate the complex biomechanical environment of the human spine. Specifically, differences in vertebral size, loading patterns, and intervertebral disc anatomy may influence outcomes such as thermal conductivity during PMMA polymerization and subsequent bone remodeling responses. To address this, future studies should consider using larger animal models, such as sheep, which possess vertebral morphology and load-bearing characteristics more comparable to those of humans. Additionally, this study did not evaluate the long-term biological responses, including chronic inflammation, fibrotic encapsulation, or potential degradation of the cement material over time. These aspects are crucial for evaluating the clinical relevance, biocompatibility, and mechanical durability of PMMA-based spinal interventions. To comprehensively assess these long-term effects, future studies should incorporate extended in vivo evaluations (e.g., 3–6 months or longer). Furthermore, dose optimization—balancing sufficient mechanical support with minimal cytotoxicity—remains to be explored to reduce the risk of adjacent tissue damage or thermal necrosis during polymerization.

Future studies should investigate combinatorial approaches using pharmacological agents such as bisphosphonates, teriparatide, or romosozumab to enhance the regenerative potential of spine-specific PMMA. Translational research using large animal models under weight-bearing conditions is also warranted to confirm these findings in clinically relevant scenarios.

## 4. Materials and Methods

### 4.1. Study Design

**Scheme 1:**
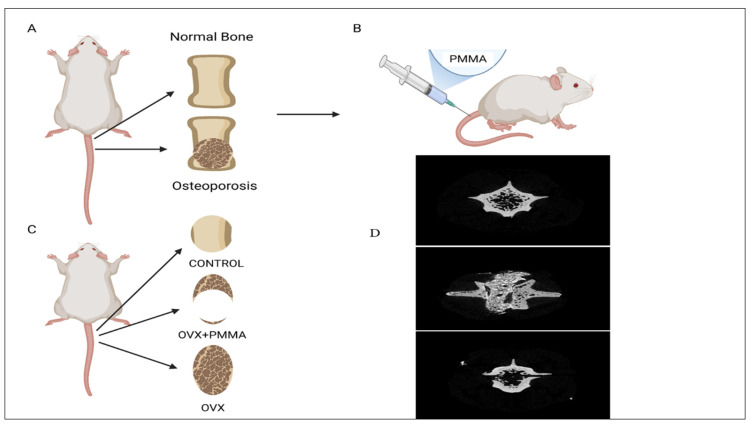
Designing a study on osteoporosis prevention and the interaction between bone and bone cement in ovariectomized rats: (**A**) Development of an osteoporotic rat model. (**B**) PVP in the osteoporotic rat model. (**C**) Images depicting various experimental groups. (**D**) Control group demonstrates normal vertebrae without issues and bone growth characteristics of spine-specific PMMA bone cement, contrasted with osteoporosis observed in the OVX group.

This figure represents an osteoporosis study using an ovariectomized (OVX) rat model (Scheme 1A). It compares normal and osteoporotic bone structures to highlight the effects of osteoporosis. PMMA is injected into the rats to observe potential therapeutic outcomes (Scheme 1B). Different experimental groups, including the Control, OVX, and OVX + PMMA, are analyzed for bone structure variations (Scheme 1C). The control group displays normal vertebrae with no abnormalities, along with typical bone growth patterns associated with spine-targeted PMMA bone cement, in contrast to the osteoporotic changes seen in the OVX group (Scheme 1D).

A schematic drawing of the main elements of the osteoporotic rat model and the clinical study is presented in Scheme 1.

Spine-specific PMMA bone cement (Mendec Spine Cement; Tecres SPA, Verona, Italy) is commercially available and has been approved for use in PVP and BKP. The compositions of spine-specific PMMA bone cement are presented in Table 1. For each bone cement, the powder and liquid (in a ratio of 2.20/2.60 g mL^−1^) were thoroughly mixed to obtain a homogeneous dough, which was then injected into the caudal vertebrae. We have included the composition details of Spine-Specific PMMA and PMMA in Table 1 and Table 2 respectively.

### 4.2. Preparation of the Rat’s Osteoporosis Model

Twenty-four female Sprague–Dawley rats (8 weeks old, approximately 260 g) were housed under standard conditions with ad libitum access to food and water. The animals were randomly assigned into three groups (*n* = 8 per group): the Control (untreated), OVX (ovariectomized), and OVX + PMMA (ovariectomized with spine-specific PMMA injection) (Table 4). All experimental procedures were approved by the Institutional Animal Care and Use Committee (IACUC; Approval No. CMCDJ-AP-2024-006, 2024-12-05).

A bilateral ovariectomy was performed under isoflurane anesthesia. Postoperative care included gentamycin (2 mL/kg) to prevent infection In the OVX + PMMA group, spine-specific PMMA (Mendec Spine Cement; Tecres SPA, Verona, Italy) was injected into the caudal vertebrae (C3–C5) once the cement reached a dough-like consistency. The components (powder/liquid ratio = 2.2:2.6 g/mL) were mixed according to the manufacturer’s instructions.

### 4.3. Osteoporotic Rat Model

Sixteen osteoporotic female Sprague–Dawley rats were randomly allocated into two groups of equal size: the OVX group and the group receiving spine-specific PMMA bone cement. Anesthesia was administered using isoflurane (30 mg/kg) via the ear vein. The rats were positioned prone, and the surgical area was disinfected following standard protocols. Injection sites were identified near the caudal vertebral body, and small incisions were made to allow for needle insertion. Once the desired location was reached, the cannula was filled with bone cement material. After the material had solidified, the needle was carefully withdrawn. All surgical procedures were completed without any rat mortality, and only minimal bleeding or tissue injury was observed.

### 4.4. Dual Energy X-Ray Absorptiometry

Dual-energy X-ray absorptiometry (DXA) was used to evaluate the bone mineral density (BMD) and bone mineral content (BMC) at predefined regions of interest (ROI-1: femoral shaft; ROI-2: caudal vertebrae C2–C5) using an InAlyzer (Medikors, Republic of Korea).

### 4.5. Micro-CT Imaging of the Treated Caudal Vertebral Body

Twelve weeks after PVP treatment, the rats (*n* = 8) were euthanized, and their caudal vertebrae were collected for analysis. All specimens underwent micro-computed tomography (micro-CT) scanning. The scans were reconstructed using a consistent protocol with a data analysis system (SkyScan, Kontich, Belgium). An industrial-grade micro-CT scanner was utilized, operating at an accelerating voltage of 225 kV with a spatial resolution of 4 μm, to capture high-resolution images of the caudal vertebral bodies. From each vertebra, cylindrical regions measuring 1.5 mm in both diameter and height were extracted for quantitative evaluation of the bone parameters, including the bone volume fraction (BV/TV, %), trabecular thickness (mm), and trabecular number (1/mm).

### 4.6. Quantitative Polymerase Chain Reaction (qPCR)

After crushing the bones using liquid nitrogen, total RNA was extracted with the RNeasy Mini Kit (Qiagen, Hilden, Germany). Complementary DNA (cDNA) synthesis was performed using the Reverse Transcription Premix for qPCR (Thermo Fisher Scientific, Korea Co., Ltd.). The relative mRNA expression levels of bone-related genes, including alkaline phosphatase (ALP), Runx2, osteocalcin (OCN), tartrate-resistant acid phosphatase (ACP-5), cathepsin K (CAT-K), and matrix metallopeptidase 9 (MMP-9), were measured using the quantitative polymerase chain reaction (qPCR). The analysis was carried out using the Applied Biosystems 7500 Fast Quantitative PCR System (Thermo Fisher Scientific, Waltham, MA, USA) in conjunction with the Thunderbird SYBR Green PCR Mix (TOYOBO, Osaka, Japan). The primer sequences used for amplification are detailed in Table 3.

### 4.7. Histological Staining and Imaging

Tissue sections were first deparaffinized using xylene and rehydrated through a descending ethanol gradient. Following a 5 min rinse in distilled water, antigen retrieval was carried out using proteinase K treatment. After another wash in distilled water, a blocking buffer was applied to inhibit nonspecific antibody binding. The sections were then incubated overnight at 4 °C with primary antibodies. These included mouse monoclonal anti-alkaline phosphatase (ALP) (1:500; Abcam, Waltham, MA, USA), rabbit monoclonal anti-RUNX2 (1:100; Novus Biologicals, Centennial, CO, USA), and mouse monoclonal anti-osteocalcin (OCN) (1:500; Cell Signaling Technology, Danvers, MA, USA).

The next day, the sections were treated with peroxidase-conjugated secondary antibodies (anti-mouse or anti-rabbit) using the VECTASTAIN Elite ABC Kit (Vector Laboratories, Newark, CA, USA) for 30 min at room temperature. Enzymatic activity was detected using the HRP-based ImmPACT NovaRED Substrate kit (Vector Laboratories, Newark, CA, USA), with an incubation time of 15 min. Mayer’s hematoxylin was used for counterstaining, followed by graded dehydration. Finally, coverslips were placed on the slides, which were then examined under an OLYMPUS BX53 U-CMAD3 microscope (T7, Tokyo, Japan).

### 4.8. Western Blotting

The tissue from the metaphysis of the mouse femur was homogenized in RIPA buffer (Meridian Rd., Rockford, IL, USA) supplemented with 1% protease and phosphatase inhibitors (GenDEPOT). The homogenate was centrifuged at 12,000 rpm, and the resulting supernatant was collected. The protein concentration was quantified using the Bradford 1× Dye Reagent (Bio-Rad, Berkeley, CA, USA). Protein samples were then denatured by boiling at 100 °C for 5 min. For electrophoresis, 10 μg of protein per sample was loaded onto a 10% SDS-polyacrylamide gel, followed by transfer to PVDF membranes. The membranes were blocked with bovine serum albumin (BSA; LPS Solution, Daejeon, Republic of Korea) for 1 h at room temperature to prevent nonspecific binding. They were then incubated overnight at 4 °C with primary antibodies specific to alkaline phosphatase (ALP), RUNX2, osteocalcin (OCN), and Beta-Actin (all from Santa Cruz Biotechnology, Dallas, TX, USA, except RUNX2 from Cell Signaling Technology, Massachusetts, USA).

After washing with TBST buffer, the membranes were treated with appropriate HRP-conjugated secondary antibodies (Santa Cruz Biotechnology, USA). Protein bands were visualized using enhanced chemiluminescence and imaged with an automated chemiluminescence detection system (Jun Yi Dong Fang, JY-MINI610, Beijing, China).

### 4.9. Statistical Analysis

Data were expressed as the mean ± standard deviation, and ONE-way ANOVA and *t*-test were used to investigate the changes in the effects of the CONT, OVX + PMMA, and OVX groups after twelve weeks. Eight rats were used for the CONT group, and sixteen rats for the OVX + PMMA and OVX groups. Statistical significance was assessed with significance levels of *p* < 0.05, *p* < 0.01, and *p* < 0.001 (the significance levels are indicated by * *p* < 0.05, ** *p* < 0.01, and *** *p* < 0.001) using GraphPad Prism 10.5.1 (GraphPad Software, Inc., La Jolla, CA, USA).

## 5. Conclusions

Our results indicate that spine-specific PMMA may offer dual benefits: mechanical support and thermal stimulation-driven osteogenesis. This localized bioactivity enhances bone regeneration in osteoporotic conditions and may represent a promising approach for managing vertebral fragility. Further optimization and long-term evaluation are required to ensure clinical efficacy and safety.

**Table 4:** Experimental Group Design.

Group	Description	*n*
Control	No surgery or treatment	8
OVX	Ovariectomized + Defect, no PMMA	8
OVX + PMMA	Ovariectomized + Spine-Specific PMMA injection	8

Schematic of the study design and injection site. PMMA injection was performed in the caudal vertebrae (C3–C5) 8 weeks after ovariectomy. Bone quality was evaluated 12 weeks post-treatment using DXA, Micro-CT, qPCR, IHC, and Western blot.

## Figures and Tables

**Table 1:** Mixing compositions of spine-specific PMMA bone cement.

Powder Components	20 g Container of Sterile Powder	Liquid Components	9.2 g Phial of Sterile Liquid
Methyl Methylcrylate Polymer	66.00% *w*/*w*	Methylmethacrylate	99.10% *w*/*w*
Barium sulfate	29.00% *w*/*w*	N-N-dimethyl-p-toluidine	0.90% *w*/*w*
Benzoyl peroxide	2.00% *w*/*w*	Hydroquinone	75 ppm

**Table 2:** Mixing compositions of PMMA bone cement.

Sample	Powder (g)				Liquid (mL)	
	Poly(methyl methacrylate-co-methyl acrylate)	BaSO_4_/ZrO_2_	Benzoyl peroxide	Methyl methacrylate	N,N-dimethyl-p-toluidine	Hydroquinone
PMMA cement	13.5	6	0.5	9.91	0.09	75 ppm

**Table 3:** Ct method. The ∆Ct value was determined by subtracting the Ct value of the reference gene (Actin) from the Ct value of the target gene (∆Ct = Ct_target gene − Ct_GAPDH). The fold change in mRNA expression, represented as 2^−∆∆Ct^, was then calculated to determine the relative mRNA levels compared to the control group.

Genes	Forward	Reverse
GAPDH	5′GTATCGGACGCCTGGTTAC3′	5′CTTGCCGTGGGTAGAGTCAT3′
ACP-5	5′TCCCCAATGCCCCATTC3′	5′CGGTTCTGGCGATCTCTTTG3′
MMP-9	5′AAAGACCTGAAAACCTCCAACCT3′	5′GCCCGGGTGTAACCATAGC3′
CAT-K	5′GGCTGTGGAGGCGGCTAT3′	5′AGAGTCAATGCCTCCGTTCTG3′
ALP	5′ACCTAGACACAAGCACTCC3′	5′GCCTCCTTCCACTAGCAAGA3′
RUNX2	5′TCCCAGTATGAGAGTAGGTGTCC3′	5′GGCTCAGATAAGGGTAAGAC3′
OCN	5′CAGCCTTCATGTCCAAGCAG3′	5′TAGAGACCACCCAGCACAAG3′

## Data Availability

Datasets are available through the corresponding author upon reasonable request.
